# Nicotine Elicits Convulsive Seizures by Activating Amygdalar Neurons

**DOI:** 10.3389/fphar.2017.00057

**Published:** 2017-02-09

**Authors:** Higor A. Iha, Naofumi Kunisawa, Saki Shimizu, Kentaro Tokudome, Takahiro Mukai, Masato Kinboshi, Akio Ikeda, Hidefumi Ito, Tadao Serikawa, Yukihiro Ohno

**Affiliations:** ^1^Laboratory of Pharmacology, Osaka University of Pharmaceutical SciencesOsaka, Japan; ^2^Department of Epilepsy, Movement Disorders and Physiology, Graduate School of Medicine, Kyoto UniversityKyoto, Japan; ^3^Department of Neurology, Graduate School of Medicine, Wakayama Medical UniversityWakayama, Japan

**Keywords:** nicotine, convulsive seizures, nicotinic acetylcholine receptors, amygdala, Fos expression

## Abstract

Nicotinic acetylcholine (nACh) receptors are implicated in the pathogenesis of epileptic disorders; however, the mechanisms of nACh receptors in seizure generation remain unknown. Here, we performed behavioral and immunohistochemical studies in mice and rats to clarify the mechanisms underlying nicotine-induced seizures. Treatment of animals with nicotine (1–4 mg/kg, i.p.) produced motor excitement in a dose-dependent manner and elicited convulsive seizures at 3 and 4 mg/kg. The nicotine-induced seizures were abolished by a subtype non-selective nACh antagonist, mecamylamine (MEC). An α7 nACh antagonist, methyllycaconitine, also significantly inhibited nicotine-induced seizures whereas an α4β2 nACh antagonist, dihydro-β-erythroidine, affected only weakly. Topographical analysis of Fos protein expression, a biological marker of neural excitation, revealed that a convulsive dose (4 mg/kg) of nicotine region-specifically activated neurons in the piriform cortex, amygdala, medial habenula, paratenial thalamus, anterior hypothalamus and solitary nucleus among 48 brain regions examined, and this was also suppressed by MEC. In addition, electric lesioning of the amygdala, but not the piriform cortex, medial habenula and thalamus, specifically inhibited nicotine-induced seizures. Furthermore, microinjection of nicotine (100 and 300 μg/side) into the amygdala elicited convulsive seizures in a dose-related manner. The present results suggest that nicotine elicits convulsive seizures by activating amygdalar neurons mainly via α7 nACh receptors.

## Introduction

Nicotine, an alkaloid derived from leaves of *Nicotiana* species, is the primary active compound of tobacco products (Saitoh et al., 1985). Acute intoxication with nicotine shows two phases of symptoms; early phase symptoms including nausea, vomiting (Forrester, 1979; Bramley and Goulding, 1981; Lavoie and Harris, 1991; Rizzi et al., 1991), headache (Woolf et al., 1997), tremors (Shiffman et al., 1983) and seizures (Dixit et al., 1971; Miner and Collins, 1989; Singer and Janz, 1990; Woolf et al., 1996; Murphy et al., 2006), and delayed phase symptoms including CNS depression and coma (Frank et al., 1995; Murphy et al., 2006). In addition, nicotine has a variety of pharmacological actions including antidepressant effects (Vieyra-Reyes et al., 2008; Mineur and Picciotto, 2010; Haj-Mirzaian et al., 2015), cognitive enhancement (Stein et al., 1998; Swan and Lessov-Schlaggar, 2007; Wood et al., 2016), positive reinforcement (addictive effects) (Stein et al., 1998; Le Foll and Goldberg, 2009; Besson et al., 2012; Harrington et al., 2016), and motor excitement (Miner and Collins, 1989; Swan and Lessov-Schlaggar, 2007; Le Foll and Goldberg, 2009; Besson et al., 2012; Wood et al., 2016).

The diverse actions of nicotine are mediated by nACh receptors, which consist of a variety combinations of α (α1–α10), β (β1–β4) and other (δ, γ, 𝜀) subunits, forming ligand-gated pentameric cation channels (Gotti et al., 2006; Dani and Bertrand, 2007; Faure et al., 2014). Specifically, neural nACh receptor subtypes are constructed from combinations of 9 α (α2–α10) and 3 β (β2–β4) subunits. Among them, homomeric α7 and heteromeric α4β2 nACh receptors are the most characterized and abundant subtypes in the brain, whereas α3β4 nACh receptors mainly function as peripheral ganglionic nACh receptors (Gotti et al., 2006; Dani and Bertrand, 2007; Taly et al., 2009; Colombo et al., 2013; Faure et al., 2014).

Since nicotine evokes motor excitement including Straub tail, tremors and convulsive seizures (Miner and Collins, 1989; Swan and Lessov-Schlaggar, 2007; Le Foll and Goldberg, 2009; Besson et al., 2012; Wood et al., 2016), nACh receptors are implicated in the pathogenesis of epileptic and movement disorders. Indeed, previous studies showed that genetic polymorphisms of α4, β2 and/or α7 subunits of nACh receptors are involved in various epileptic disorders, including idiopathic generalized epilepsy (e.g., epilepsy with generalized tonic-clonic seizures, childhood absence epilepsy, juvenile absence epilepsy and juvenile myoclonic epilepsy; Elmslie et al., 1997; Helbig et al., 2009; Endris et al., 2010; Liao et al., 2011; Rozycka et al., 2013b) and partial epilepsy (e.g., autosomal dominant nocturnal frontal lobe epilepsy and benign epilepsy of childhood with centrotemporal spikes; Steinlein et al., 1995; Bertrand et al., 2005; Pidoplichko et al., 2013; Rozycka et al., 2013a). However, the role and mechanisms of nACh receptors in seizure generation and epileptogenesis are still unknown.

Fos protein, an immediate early gene product, is widely used as a biological marker of neural excitation in neuropharmacology research (Morgan et al., 1987; Herrera and Robertson, 1996; Ohno et al., 2011; Iha et al., 2016). Specifically, mapping analysis of Fos expression is a useful method to identify brain regions linked to disease conditions (e.g., pain, epilepsy, and emotional disorders) and to various drug treatments. In the present study, therefore, we performed behavioral and Fos-immunohistochemical studies to delineate the mechanisms underlying nicotine-induced seizures in rodents.

## Materials and Methods

### Animals

Male ddY mice (Japan SLC, Shizuoka, Japan) weighing 25–35 g and male SD rats (Japan SLC, Shizuoka, Japan) weighting 250–300 g were used. The animals were kept in air-conditioned rooms under a 12 h light/dark cycle (light on: 8:00 a.m.) and allowed *ad libitum* access to food and water. The housing conditions and animal care methods complied with the Guide for the Care and Use of Laboratory Animals of the Ministry of Education, Science, Sports and Culture of Japan. The experimental protocols were approved by the Experimental Animal Research Committee at Osaka University of Pharmaceutical Sciences.

### Behavioral Evaluation

Animals were intraperitoneally injected with nicotine (1–4 mg/kg) or saline (vehicle) and placed in an individual observation box (25 cm × 42 cm × 20 cm). Nicotine-induced behavioral excitement was evaluated over 15 min after the nicotine injection using a six point-ranked score (0: no effect; 1: mild head tremor and Straub tail; 2: apparent tremors in extended regions, 3: severe tremors with wild running; 4: clonic seizures; 5: tonic or tonic-clonic seizures) modified from previous reports (Kunisawa et al., 2016; Tokudome et al., 2016). Incidence of convulsive seizures was judged as positive when scores were 4 or higher. In the experiments using nACh receptor antagonists, a subtype non-selective nACh antagonist, MEC (1 mg/kg), a specific α7 nACh antagonist, MLA (10 mg/kg), a specific α4β2 nACh antagonist, DHβE (5 mg/kg) or saline (vehicle) was intraperitoneally injected 15 min before the nicotine treatment. The dosage of nACh antagonists was set to a level that sufficiently antagonized the respective nACh receptors in previous studies [MEC: (Gomita et al., 1989), DHβE: (Blondel et al., 2000), and MLA: (Blondel et al., 2000; Kim et al., 2011; Liu, 2014)].

### Analysis of Fos Protein Expression

Staining of Fos-IR was performed using the methods published previously (Ohno et al., 2011, 2012; Iha et al., 2016). Briefly, ddY mice were treated with a convulsive dose (4 mg/kg, i.p.) of nicotine or saline (vehicle), and brain samples were obtained 120 min after the nicotine injection under pentobarbital (80 mg/kg, i.p.) anesthesia. In some experiments, mice were pretreated with MEC (1 mg/kg) 15 min before the nicotine injection. After fixation with 4% formaldehyde solution, brains were cut into coronal sections (30 μm thickness) using a Microslicer (DSK-3000, Dosaka, Kyoto, Japan). Slices were incubated in the presence of 2% normal rabbit serum for 2 h and in the presence of 2% normal rabbit serum and goat c-Fos antiserum for an additional 18–36 h. The sections were then incubated with a biotinylated rabbit anti-goat IgG for 2 h and with PBS containing 0.3% hydrogen peroxide for 30 min to inactivate endogenous peroxidase. Thereafter, the sections were incubated with avidin–biotinylated horseradish peroxidase complex for 2 h.

Fos-IR was visualized by the diaminobenzidine–nickel staining method and quantified by counting the number of Fos-IR positive nuclei in the following 48 regions (Franklin and Paxinos, 2008), (1) the cerebral cortices (19 regions), mPFC, CgC, MC (1-4), SC (1-4), AIC, PirC (1-4), Apir, AuC, PRh-Ect, DLEnt, (2) the limbic regions and basal ganglia (14 regions), AcC, AcS, BLP, BMP, PMCo, MePV, MePD, CA (1-3), DG, dlST, dmST, GP, LS, (3) the epithalamic and lower brainstem regions (15 regions), MHb, LHb, PT, PV, PH, AM, CM, VM, AH, PH, DM, RPC, SNr, SNc, Sol, IO.

### Electrical Lesion Study

Electrical lesion studies were performed using SD rats as reported previously (Ohno et al., 2015; Kunisawa et al., 2016). Briefly, animals were anesthetized with pentobarbital (60 mg/kg, i.p.) and fixed in a stereotaxic frame (Narishige, SR-6, Tokyo, Japan). A bipolar concentric electrode was bilaterally inserted into the thalamus (Th; A: -1.5 mm; L: ± 0.4 mm; H: + 4.2 mm); PirC (A: +1.3 mm; L: ± 4.3 mm; H: + 7.2 mm), MHb (A: + 0.4 mm; L: ± 0.4 mm; H: + 4 mm); or amygdala (A: -3.1 mm; L: ± 4 mm; H: + 7.9 mm; Paxinos and Watson, 2007) and a direct current of 1 mA was delivered to the respective regions for 15 s. After a recovery period (2–4 days) from the surgery, animals were treated with nicotine (4 mg/kg) or vehicle, individually placed in an observation box and underwent behavioral evaluation as described previously. After the experiments, the animals were deeply anesthetized with pentobarbital (80 mg/kg, i.p.) and the brain was removed from the skull in order to check the position of each electrical lesion.

### Microinjection Study

Microinjection studies were performed using SD rats as reported previously (Shimizu et al., 2010, 2013, 2014). After the animals were fixed in a stereotaxic instrument under pentobarbital (40 mg/kg, i.p.) anesthesia, a stainless steel guide cannula was bilaterally inserted 1 mm above the amygdala (A: -3.1 mm; L: ± 4 mm; H: + 7.9 mm; Paxinos and Watson, 2007) and fixed on the skull with dental cement. After a recovery period (2–4 days), an injection cannula was inserted into the amygdala through a guide cannula and nicotine (100 or 300 μg/μL per side) was injected at a flow rate of 0.25 μL/min (Microinfusion pump KDS220; KD Scientific Inc., USA) for 4 min under freely moving conditions. The control animals were given the same volume of saline (vehicle) alone. Nicotine-induced behavioral excitement was evaluated as previously described using a six point-ranked score. After the experiment, animals were deeply anesthetized with pentobarbital (80 mg/kg, i.p.) and their brains were removed for subsequent guide cannula insertion site verification.

### Drugs

Nicotine, MEC hydrochloride, MLA citrate and DAB substrate were purchased from Sigma-Aldrich (St. Louis, MO, USA) and DHβE hydrobromide from Tocris (Bristol, UK). The primary antibody against c-Fos was purchased from Santa Cruz Biotechnology Inc. (Santa Cruz, CA, USA), and the secondary biotinylated anti-goat IgG antibody, ABC kit from Vector Laboratories (Burlingame, CA, USA). Others common laboratory reagents were also obtained from commercial sources.

### Statistical Analysis

Data are expressed as the mean ± S.E.M. Statistical significance of differences among multiple groups was determined by the Kruskal–Wallis test followed by the Steel-Dwass *post hoc* test (behavioral scores) or one-way ANOVA followed by Tukey’s *post hoc* test (Fos expression). Comparisons between only groups were determined by parametric Student’s *t*-test (Fos expression) or non-parametric Mann–Whitney’s *U* test (electrical lesion). Comparisons of seizure incidence rate were done by χ^2^ test. A *P*-value of less than 0.05 was considered statistically significant.

## Results

### Nicotine-Induced Convulsive Seizures

**FIGURE 1:**
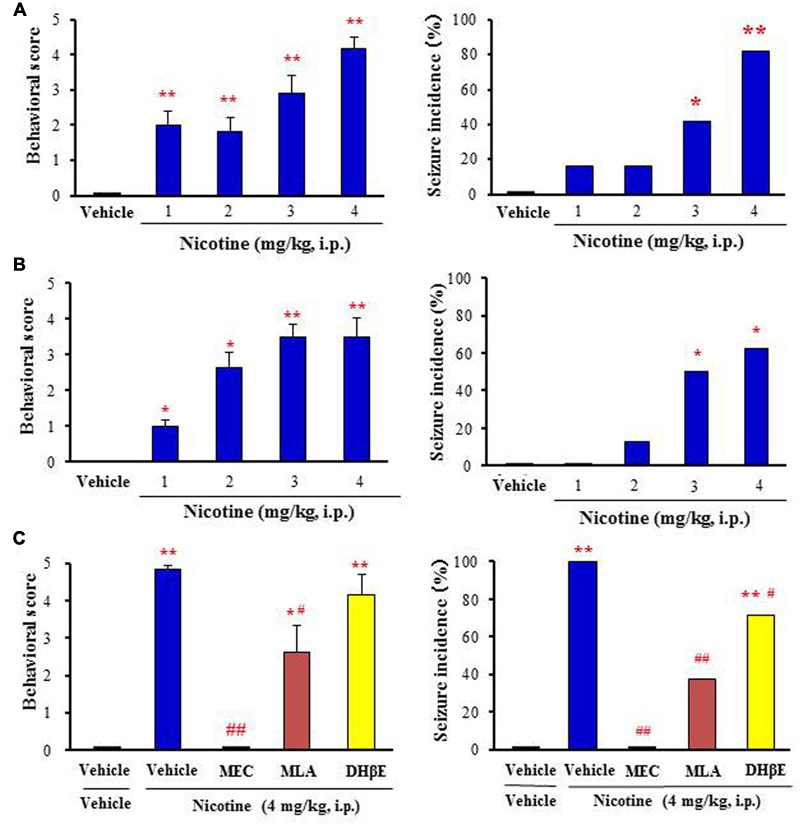
**Effects of nicotine on convulsive seizure induction in rodents. (A,B)** Nicotine-induced convulsive seizures in mice **(A)** and rats **(B)**, respectively. **(C)** Effects of nACh antagonists, MEC (non-selective; 1 mg/kg i.p.), MLA (α7 nACh antagonist; 10 mg/kg i.p.), and DHβE (α4β2 nACh antagonist; 5 mg/kg i.p.) on nicotine (4 mg/kg. i.p.)-induced seizures in mice. Behavioral scores are expressed as the mean ± S.E.M. of 7–11 animals. Seizure incidence represents the percentage of animals, which showed convulsive seizures (score 4 or 5), against total animals examined. ^∗^*P* < 0.05 and ^∗∗^*P* < 0.01; Significantly different from the control animals treated with vehicle alone (Vehicle or Vehile+Vehicle). ^#^*P* < 0.05 and ^##^*P* < 0.01; Significantly different from the value for nicotine group (Vehicle+Nicotine).

Nicotine at doses from 1 to 4 mg/kg (i.p.) dose-dependently produced motor excitement both in mice and rats, inducing Straub tail and tremor (score 1–3) at low doses (i.e., 1–2 mg/kg, i.p.) and convulsive seizures (score 4 or 5) at high doses (i.e., 3–4 mg/kg, i.p.; **Figures 1A,B**). The incidence of nicotine-induced motor excitement including seizures was normally transient and subsided within 10 min. The percentages of animals which showed clonic or tonic-clonic seizures with nicotine (4 mg/kg, i.p.) were 82 and 62.5% in mice and rats, respectively (**Figures 1A,B**).

To clarify the subtype of nACh receptors involved in nicotine-induced seizures, we tested the actions of nACh antagonists in mice. Pretreatment of animals with a subtype non-selective nACh antagonist, MEC (1 mg/kg, i.p.) markedly reduced the seizure intensity and incidence rate due to nicotine (4 mg/kg, i.p.; **Figure 1C**). An α7 nACh antagonist MLA (10 mg/kg, i.p.) also significantly inhibited nicotine-induced seizures whereas a specific α4β2 nACh antagonist DHβE (5 mg/kg, i.p.) only weakly reduced the seizure intensity and incidence (**Figure 1C**).

### Nicotine-Induced Fos Expression

**FIGURE 2:**
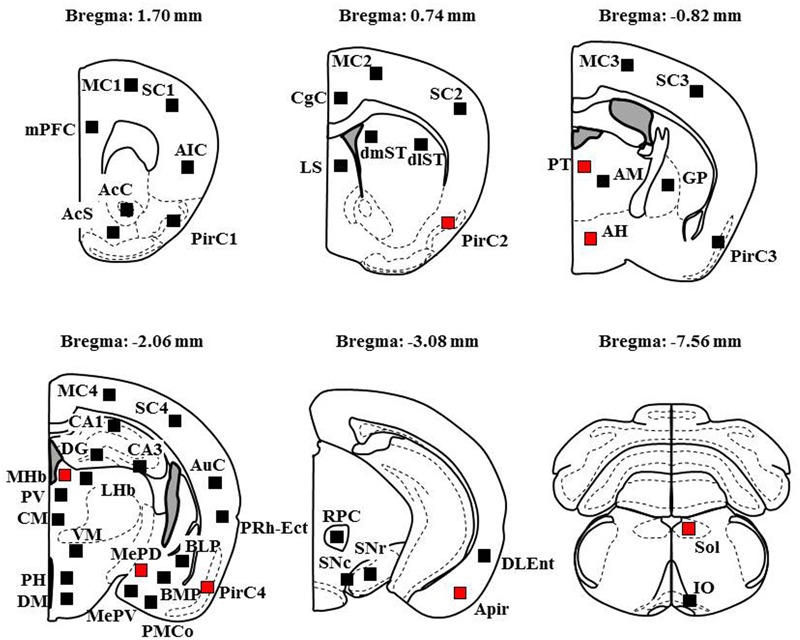
**Schematic illustrations of the brain sections selected for quantitative analysis of Fos expression.** Filled squares in each section indicate the sampling areas analyzed and red squares represent the sites which showed significant increments in Fos expression by nicotine (4 mg/kg, i.p.). Anteroposterior coordinate (distance from the bregma) is shown above each section. Analysis of the MC, SC, and PirC were performed in four different levels from Bregma (Area 1 at +1.7 mm, Area 2 at +0.74 mm, Area 3 at -0.82 m, Area 4 at -2.06 mm).

**FIGURE 3:**
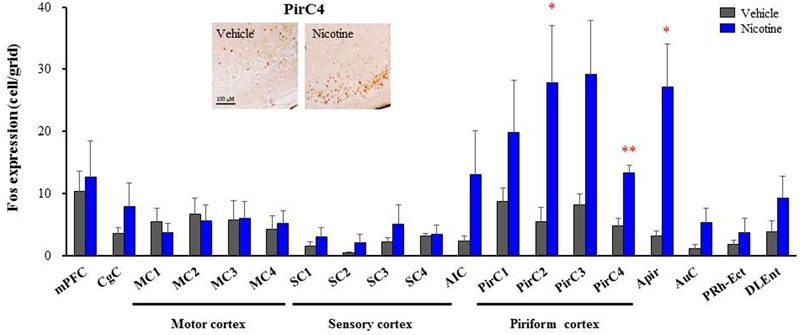
**Effects of nicotine (4 mg/kg, i.p.) on Fos expression in cortical regions in mice.** Brains were removed 2 h after the nicotine (4 mg/kg, i.p.) administration and subjected Fos-immunochemical staining. Representative photographs illustrating the Fos-IR-positive cells in the PirC4 are shown in the left top. Each column represents the mean ± S.E.M. of 5–8 mice. ^∗^*P* < 0.05, ^∗∗^*P* < 0.01; Significantly different from the control animals treated with vehicle alone (Vehicle).

**FIGURE 4:**
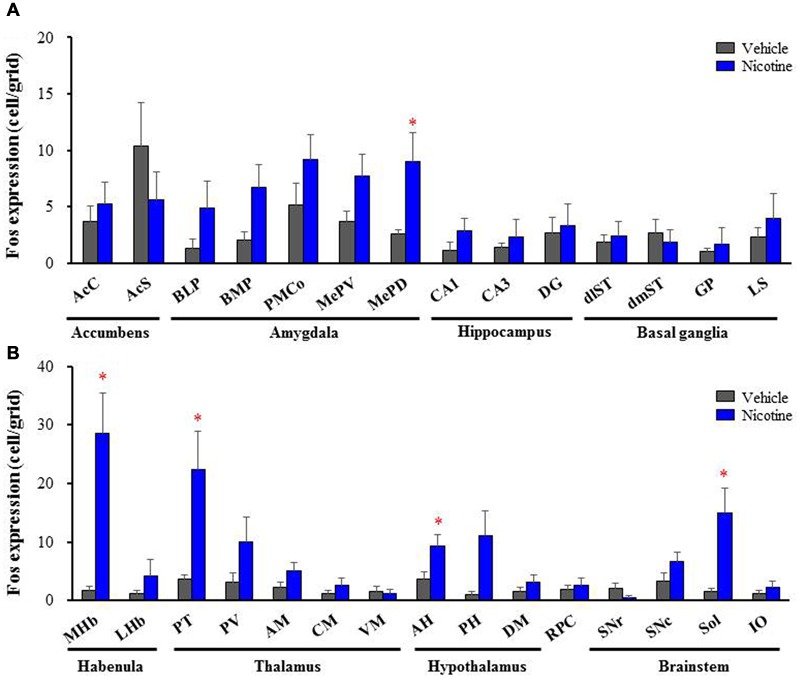
**Effects of nicotine (4 mg/kg, i.p.) on Fos expression in subcortical regions in mice. (A)** Fos expression in the limbic regions and basal ganglia. **(B)** Fos expression in the brain stem regions. Brains were removed 2 h after the nicotine (4 mg/kg, i.p.) administration and subjected Fos-immunochemical staining. Each column represents the mean ± S.E.M. of 5–8 mice. ^∗^*P* < 0.05; Significantly different from the control animals treated with vehicle alone (Vehicle).

To explore brain regions excited with nicotine-induced seizures, we analyzed the topographical expression of Fos protein, a biological marker of neural excitation, in mice. Treatment of animals with a convulsive dose (4 mg/kg, i.p.) of nicotine caused a region-specific elevation of Fos expression in 7 out of 48 brain regions examined (**Figure 2**). In the 19 cortical regions, nicotine increased Fos expression in the PirC2 [*t*(7) = 2.385, *P* = 0.050; PirC4, *t*(12) = 4.783, *P* < 0.001] and APir [*t*(10) = 3.470, *P* = 0.013] (**Figure 3**). In the 29 subcortical regions, nicotine significantly enhanced Fos expression in the amygdala, medial habenula [MHb, *t*(5) = 3.982, *P* = 0.010], paratenial thalamus [PT, *t*(6) = 2.882, *P* = 0.027], AH [*t*(10) = 2.397, AH, *P* = 0.037] and in the solitary tract nucleus [Sol, *t*(5) = 3.121, *P* = 0.025] (**Figure 4**). In the amygdala, all investigated regions showed considerably high Fos expression (about two to four times the control level) with nicotine, while it reached statistical significance only in the medial posterodorsal region [MePD, *t*(6) = 2.439, *P* = 0.048]. Other brain regions including the hippocampus, striatum, GP and substantia nigra, did not show any significant changes in Fos expression (**Figure 4**).

**FIGURE 5:**
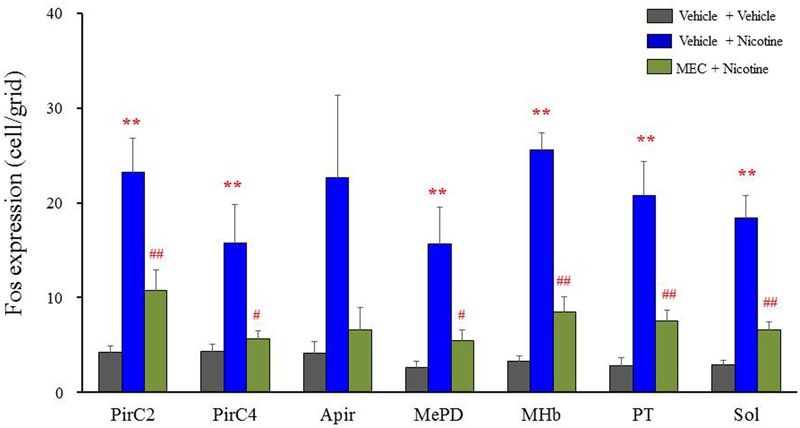
**Effects of MEC on nicotine-induced Fos expression in mice.** Animals were pretreated with MEC (1 mg/kg, i.p.) 15 min before the nicotine injection (4 mg/kg i.p.). Each column represents the mean ± S.E.M. of 5–8 mice. ^∗∗^*P* < 0.01; Significantly different from the control animals treated with vehicle alone (Vehicle + Vehicle). ^#^*P* < 0.05 and ^##^*P* < 0.01; Significantly different from the nicotine groups (Vehicle + Nicotine).

To confirm the involvement of nACh receptors, we assessed the effects of MEC on nicotine-induced Fos expression in the above seven brain regions (i.e., PirC2, PirC4, Apir, MePD, MHb, PT, and Sol). We confirmed that nicotine (4 mg/kg, i.p.) significantly elevated Fos expression in the PirC2 [*F*(2,21) = 15.880, *P* < 0.001], PirC4 [*F*(2,23) = 7.498, *P* = 0.003], MePD [*F*(2,20) = 7.771, *P* = 0.003], MHb [*F*(2,25) = 86.928, *P* < 0.001], PT [*F*(2,20) = 16.097, *P* < 0.001] and Sol [*F*(2,21) = 35.564, *P* < 0.001] (**Figure 5**). The nicotine-induced Fos expression was mostly abolished by MEC [*F*(2,21) = 15.880, PirC2, *F*(2,23) = 7.498, *P* = 0.004; PirC4, *F*(2,20) = 7.771, *P* = 0.027; MePD, *F*(2,25) = 86.928, *P* = 0.026; MHb, *F*(2,25) = 86.928, *P* < 0.001; PT, *F*(2,20) = 16.097, *P* = 0.001; and Sol, *F*(2,21) = 35.564, *P* < 0.001], indicating that nicotine-induced Fos expression is mediated by nACh receptors in these brain regions (**Figure 5**).

### Electrical Lesion Studies

**FIGURE 6:**
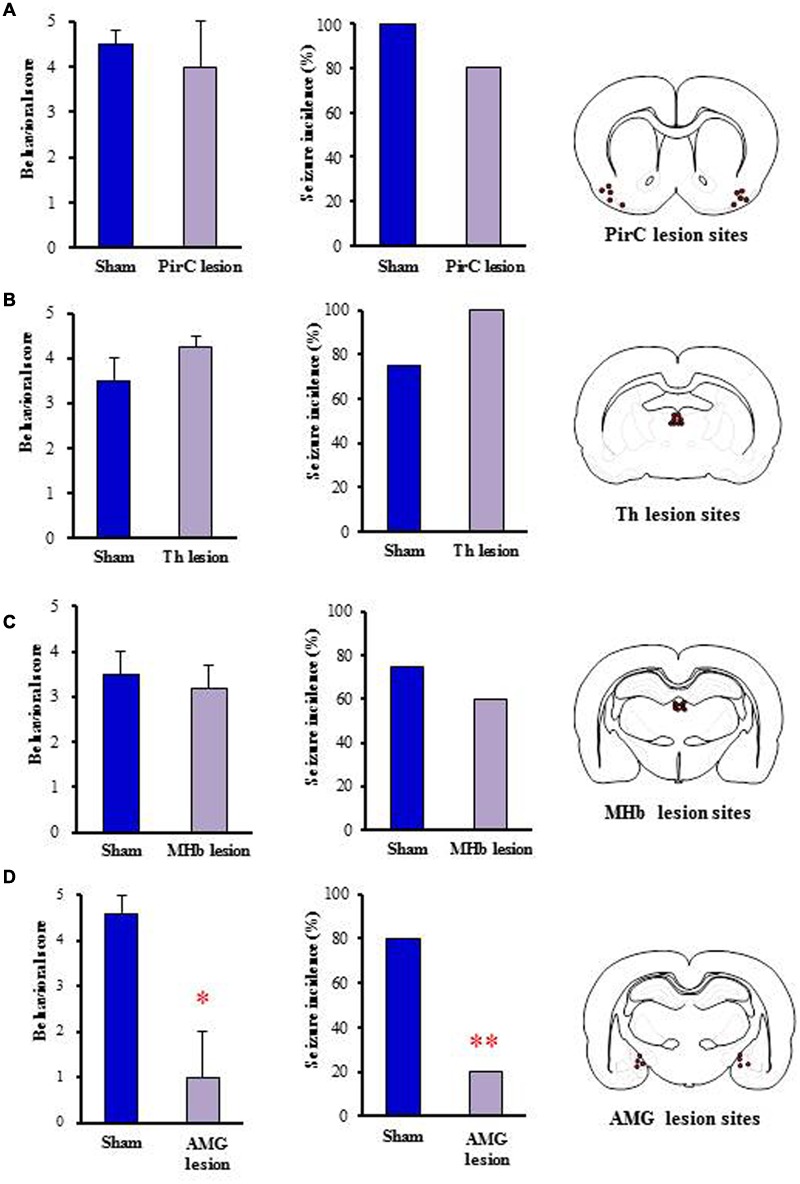
**Effects of electrical lesionings on nicotine-induced seizures in rats.** Panels show the effects of nicotine (4 mg/kg, i.p.) on seizure generation in rats after electrical lesionings at the PirC **(A)**, thalamus (Th, **B**), MHb **(C)**, and amygdala (AMG, **D**). Right panels illustrate the electrical lesion sites in Pir, Th, MHb, and AMG. Behavioral scores (left graph) are expressed as the mean ± S.E.M. of four or five animals. Seizure incidence (right graph) represents the percentage of animals, which showed convulsive seizures (score 4 or 5), against total animals examined. ^∗^*P* < 0.05 and ^∗∗^*P* < 0.01; significantly different from the Sham group.

To determine the brain regions responsible for generation of nicotine seizures, we next conducted electrical lesion studies of the sites which showed high Fos expression with nicotine in rats. The animals received electrical lesioning at the bilateral PirC, Th, MHb or amygdala 2–4 days before the nicotine-induced seizure test. Under these conditions, only the lesioning of the amygdala markedly reduced the intensity [*U*(8) = 3.000, *P* = 0.028] and the incidence (χ^2^= 0.225, *P* = 0.009) of nicotine-induced seizures. In contrast, neither lesioning of PirC, Th nor MHb affected seizure induction (**Figure 6**), suggesting that the amygdala is responsible for generation of nicotine seizures.

### Microinjection

**FIGURE 7:**
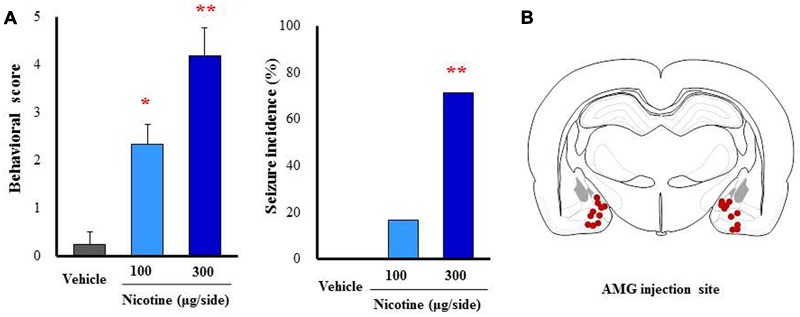
**Effects of nicotine microinjected into the amygdala (AMG) on convulsive seizure induction in rats.** Nicotine (100 or 300 μg/side) was locally injected into the bilateral AMG. **(A)** Behavioral scores (left graph) are expressed as the mean ± S.E.M. of 4–6 animals. Seizure incidence (right graph) represents the percentage of animals, which showed convulsive seizures (score 4 or 5), against total animals examined. ^∗^*P* < 0.05 and ^∗∗^*P <* 0.01; Significantly different from the control group treated with vehicle alone (Vehicle). **(B)** The injected sites of nicotine in the AMG.

To further confirm the causative role of the amygdala, we performed a microinjection study with nicotine into the amygdala. Under a freely moving condition, 100 and 300 μg/side of nicotine were injected into the bilateral amygdala. As shown in **Figure 7**, nicotine caused motor excitement (100 μg/side; χ^2^ = 13.602, df = 2, *P* = 0.0136, 300 μg/side; χ^2^ = 13.602, df = 2, *P* = 0.005) and seizure generation (300 μg/side; χ^2^= 5.76, *P* = 0.016) in a dose-related manner (**Figure 7**).

## Discussion

Nicotine has proconvulsive actions and, when overdosed, induces convulsive seizures both in humans and animals (Singer and Janz, 1990; Murphy et al., 2006; Rong et al., 2014). We confirmed that nicotine dose-dependently caused convulsive seizures in rodents (ddY mice and SD rats). The dosage (3–4 mg/kg, i.p.) of nicotine that caused convulsions was similar to those in previous reports (Miner et al., 1985; Miner and Collins, 1989), where various mouse strains were evaluated for nicotine-induced seizure sensitivity (more sensitive ST/bj mice ED_50_ = 2.34, mg/kg, i.p. and less sensitive DB mice ED_50_ = 6.16 mg/kg, i.p.).

Although several studies suggest that proconvulsive action of nicotine is mediated by α7 nACh receptors (Damaj et al., 1999; Gil et al., 2002; Dobelis et al., 2003), nACh receptors subtypes involved in nicotine-induced seizures are still uncertain. Here, nicotine-induced seizures were completely blocked by MEC, illustrating nACh receptor mediation. In addition, MLA (α7 nACh antagonist) was considerably more potent than DHβE (α4β2 nACh antagonist) in inhibiting nicotine-induced seizures. These results are consistent with previous studies (Damaj et al., 1999; Gil et al., 2002; Dobelis et al., 2003) and suggest that α7 nACh receptors play a major role in inducing nicotine seizures. We have previously reported that kinetic tremors induced by a low dose (1 mg/kg, i.p.) of nicotine are mediated by α7 nACh receptors, whereas α4β2 nACh receptors are negligibly involved in tremor induction (Kunisawa et al., 2016). Therefore, α7 nACh receptors are likely to play a key role in producing motor excitations (e.g., tremor and seizure generation) with nicotine. However, we cannot completely deny a possibility that α4β2 nACh receptors are partly involved in nicotine-induced seizures since DHβE slightly reduced the seizure induction, which is consistent with the previous finding that i.c.v. injection of DHβE reduced nicotine seizures by about 15% (Damaj et al., 1999).

Fos protein expression is widely used as a marker of neural activation to explore the brain regions linked to disease conditions (e.g., epilepsy, essential tremors) and drug responses (Morgan et al., 1987; Kovacs, 1998; Hoffman and Lyo, 2002; Ohno et al., 2008, 2009, 2011; Okuno, 2011; Tatara et al., 2015; Iha et al., 2016). We previously demonstrated that a low dose (1 mg/kg, i.p.) of nicotine, which reportedly induces cognitive enhancement (Swan and Lessov-Schlaggar, 2007), antidepressant effects (Vieyra-Reyes et al., 2008; Mineur and Picciotto, 2010) and positive reinforcement (Harrington et al., 2016), as well as kinetic tremor (Kunisawa et al., 2016), region-specifically elevated Fos expression in four brain regions; the PirC, MHb, Sol, and IO. In the present study, a convulsive dose (4 mg/kg, i.p.) of nicotine further increased Fos expression in extended regions, the amygdala and parts of the diencephalon (thalamus and hypothalamus). Thus, these regions excited by nicotine seemed to be related to seizure induction. In addition, an electrical lesion study revealed that only the amygdala lesion, but not the PirC, Th, or MHb lesions, suppressed nicotine-induced seizures, suggesting that the amygdala is the causative site for the induction of nicotine seizures. This possibility was further supported by the fact that microinjected nicotine into the amygdala elicited convulsive seizures. The amygdala is well known to be involved in seizure generation and epileptogenesis (Gloor, 1992; Pitkanen et al., 1998; Morimoto et al., 2004; Aroniadou-Anderjaska et al., 2008). In addition, previous *in situ* hybridization and autoradiography studies revealed that α7 nACh receptors are highly expressed in the amygdala (Arimatsu et al., 1978; Han et al., 2003; McCullumsmith et al., 2004; Terry et al., 2005; Gozzi et al., 2006; Klein and Yakel, 2006; Weiss et al., 2007; Viel et al., 2012; Mendez et al., 2013). Therefore, it is most likely that the amygdala, especially the medial amygdala (e.g., MePD), is the primary foci of seizure generation by nicotine. However, we cannot limit the causative site to the medial amygdala in the amygdala since other amygdaloid nuclei (e.g., BLP and BMP) also showed considerably high Fos expression with nicotine and are known to receive dense cholinergic input from the basal forebrain (Woolf et al., 1984; Emre et al., 1993; Pidoplichko et al., 2013).

Although genetic polymorphisms of the gene (*CHRNA7*) encoding the α7 nACh receptor subunit are known to be involved in various epileptic disorders in humans, including idiopathic generalized epilepsy, childhood absence epilepsy, juvenile myoclonic epilepsy and benign epilepsy of childhood with centrotemporal spikes (Elmslie et al., 1997; Helbig et al., 2009; Endris et al., 2010; Liao et al., 2011), functional role and mechanisms of α7 nACh receptors in modulating seizure generation and/or epileptogenesis are still unknown. A line of studies showed that microdeletion of chromosome 15q13.3 including *CHRNA7* causes severe mental retardation, seizures and facial and/or digital dysmorphisms. This evidence implies that α7 nACh receptors are involved in the pathogenesis of mental illness (e.g., autism and schizophrenia) and negatively regulate seizure generation (Sharp et al., 2008; Helbig et al., 2009; Sinkus et al., 2015). Nonetheless, the present results suggest that excessive stimulation of α7 nACh receptors elicits convulsive seizures by activating the amygdala neurons, which are implicated in seizure generation not only due to nicotine intoxication, but also that caused by epileptic diseases. Therefore, a gain-of-function mutation and/or copy number polymorphism (e.g., duplication and triplication) of *CHRNA7* may be associated with epileptic disorders. Indeed, patients with duplication and triplication of *CHRNA7* (15q13.3 gains) have been shown to exhibit neuropsychiatric phenotypes including epileptic seizures (Miller et al., 2009; Soler-Alfonso et al., 2014; Gillentine and Schaaf, 2015). Further studies are required to delineate the role and clinical relevance of the α7 nACh receptor in the pathogenesis of epileptic disorders.

## Conclusion

We performed behavioral and Fos-immunohistochemical studies in rodents to clarify the mechanisms underlying nicotine-induced seizures. Treatment of animals with nicotine produced motor excitement and elicited convulsive seizures at 3 and 4 mg/kg. MEC and an α7 nACh antagonist, MLA, effectively blocked the nicotine seizures, but an α4β2 nACh antagonist, DHβE, did so only weakly. In addition, Fos expression analysis revealed that that a convulsive dose (4 mg/kg) of nicotine region-specifically activated neurons in the PirC, amygdala, MHb, PT, AH and Sol, among which electric lesioning of the amygdala specifically inhibited nicotine seizure generation. Furthermore, microinjections of nicotine into the amygdala evoked convulsive seizures in a dose-related manner. The present results strongly suggest that nicotine elicits convulsive seizures by activating amygdalar neurons mainly via α7 nACh receptors.

## Author Contributions

YO designed research. HAI, NK, SS, KT, TM, MK, and YO performed experiments. HAI, NK, SS, AI, HI, TS, and YO analyzed and discussed data. HAI, NK, TS, and YO wrote the paper.

## Conflict of Interest Statement

The authors declare that the research was conducted in the absence of any commercial or financial relationships that could be construed as a potential conflict of interest.

## Abbreviations

AcC: core region of nucleus accumbens
AcS: shell region of nucleus accumbens
AH: anterior hypothalamus
AIC: agranular insular cortex
AM: anteromedial thalamic nucleus
Apir: amygdalopiriform transition area
AuC: auditory cortex
BLP: basolateral amygdaloid nucleus
BMP: basomedial amygdaloid nucleus
CA: cornu ammonis area of hippocampus
CgC: cingulate cortex
CM: centromedial thalamic nucleus
DG: dentate gyrus of the hippocampus
DHβE: dihydro-β-erythroidine
DLEnt: dorsolateral entorhinal cortex
dlST: dorsolateral striatum
DM: dorsomedial hypothalamic nucleus
dmST: dorsomedial striatum
Fos-IR: Fos-immunoreactivity
GP: globus pallidus
IO: inferior olive
LHb: lateral habenular nucleus
LS: lateral septum
MC: motor cortex
MEC: mecamylamine
MePD: medial posterodorsal amygdaloid nucleus
MePV: medial posteroventral amygdaloid nucleus
MHb: medial habenular nucleus
MLA: methyllycaconitine
mPFC: medial prefrontal cortex
nACh: nicotinic acetylcholine
PH: posterior hypothalamus
PirC: piriform cortex
PMCo: posteromedial cortical amygdaloid nucleus
PRh-Ect: perirhinal-ectorhinal cortex
PT: paratenial thalamic nucleus
PV: paraventricular thalamic nucleus
RPC: parvocellular part of the red nucleus
SC: sensory cortex
SNc: substantia nigra pars compacta
SNr: substantia nigra pars reticulata
Sol: nucleus solitary tract
VM: ventromedial thalamic nucleus
